# Quantum synchronization in disordered superconducting metamaterials

**DOI:** 10.1038/srep43657

**Published:** 2017-03-02

**Authors:** M. V. Fistul

**Affiliations:** 1 Theoretische Physik III, Ruhr-Universität Bochum, Universität str. 150, D-44801 Bochum, Germany; 2 National University of Science and Technology MISiS, Leninsky prosp. 4, Moscow, 119049, Russia; 3 Russian Quantum Center, Skolkovo, Moscow region, 143025, Russia

## Abstract

I report a theoretical study of collective coherent quantum-mechanical oscillations in disordered superconducting quantum metamaterials (SQMs), i.e. artificial arrays of interacting qubits (two-levels system). An unavoidable disorder in qubits parameters results in a substantial spread of qubits frequencies, and in the absence of electromagnetic interaction between qubits these individual quantum-mechanical oscillations of single qubits manifest themselves by a large number of small resonant dips in the frequency dependent transmission of electromagnetic waves, |*S*_21_(*ω*)|^2^. We show that even a weak electromagnetic interaction between adjacent qubits can overcome the disorder and establish completely or partially *synchronized* quantum-mechanical dynamic state in the disordered SQM. In such a state a large amount of qubits displays the collective quantum mechanical oscillations, and this collective behavior manifests itself by a few giant resonant dips in the |*S*_21_(*ω*)|^2^ dependence. The size of a system *r*_0_ showing the collective (synchronized) quantum-mechanical behavior is determined in the one-dimensional SQMs as *r*_0_ ≃ *a* [*K*/*δ*Δ]^2^, where *K, δ*Δ, *a* are the effective energy of nearest-neighbor interaction, the spread of qubits energy splitting, and the distance between qubits, accordingly. We show that this phenomenon is mapped to the Anderson localization of spinon-type excitations arising in the SQM.

Superconducting quantum metamaterials (SQMs) are novel artificially prepared solid state structures whose electrodynamic properties are governed by peculiar interplay of classical Maxwell electrodynamics and quantum-mechanical laws^1^,^2^,^3^,^4^,^5^,^6^,^7^,^8^,^9^,^10^. Most of SQMs consists of an array of interacting superconducting qubits (two-level systems), e.g. charge qubits^11^, flux qubits^12^, transmons^13^ etc.

Numerous experiments on the propagation of electromagnetic waves through the SQM^1^,^5^,^6^,^7^,^14^ have allowed one to observe an excitation of coherent quantum-mechanical oscillations between two states of single qubits. This intrinsically quantum-mechanical macroscopic phenomenon manifests themselves by sharp resonant dips in the frequency dependent transmission coefficient |*S*_21_(*ω*)|^2^ of electromagnetic waves propagating in the transmission line electromagnetically (inductively or capacitively) coupled to the SQM^1^,^5^,^6^,^7^,^13^,^14^,^15^,^16^,^17^. Such measurement setup is presented schematically in Fig. 1. In a simplest case of an “ideal” SQM consisting of an array of *non-interacting identical* qubits the transmission coefficient, |*S*_21_(*ω*)|^2^, displays a single dip on the frequency *ω* = Δ/*ħ*, where each qubit is characterized by the energy level difference Δ. A small width of such dip is determined by various dissipative and decoherent processes.

However, in imperfect SQMs an unavoidable disorder in superconducting qubits parameters is always present, and it results in a substantial spread of energy level differences Δ_*i*_. In this case it is plausible to suppose that the individual coherent quantum-mechanical oscillations of single qubits occur in the SQM, and the |*S*_21_(*ω*)|^2^ has to display a large amount of small resonant dips up to the value *N*, where *N* is a number of qubits in the SQM^15^,^16^. Indeed, in ref. 7 the frequency dependent transmission coefficient |*S*_21_(*ω*)|^2^ of the SQM containing 7 non-interacting qubits has been measured, and 7 different small resonant dips in the |*S*_21_(*ω*)|^2^ have been observed. Surprisingly in other experiments carried out on diverse SQMs a small number (one or two) of giant resonant dips has been observed in spite of the presence of large number of various qubits with different parameters^1^,^17^. However, the physical conditions necessitating to observe such *collective (synchronized) quantum-mechanical* behavior are not clear at this moment.

A key point allowing one to resolve such a puzzle is *electromagnetic interactions* between qubits. Two types of interactions can be realized in the SQMs: the nearest-neighbor interaction that is due to direct inductive or capacitive electromagnetic interaction between adjacent qubits^18^, and/or the long-range electromagnetic interaction arising due to consequent emission, propagation and absorbtion of virtual photons in the low-dissipative resonator coupled to the array of qubits^10^,^15^,^19^. Both interactions depend on the coupling coefficient *g*, where *g* is determined by mutual inductance between qubits or a single qubit and the resonator. The direct nearest-neighbor interaction is proportional to *g*, and the strength of long-range interaction is proportional to *g*^2^. Although the latter interaction is rather weak, the long-range character of such interaction allows to establish the collective effects. E.g. in ref. 15 the collective ac-Stark shift of qubit frequencies has been theoretically predicted. Moreover, this ac-Stark frequency shift is resonantly enhanced as the frequency of resonator *ω*_*r*_ coincides with the average value of qubit frequencies 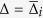.

In this Article we consider the case of inductive interaction between adjacent qubits, and we show that such interaction between qubits can overcome the disorder and establish collective synchronized quantum-mechanical oscillations characterized by just few frequencies. A weak inductive interaction results in a slight increase of energy of coupled qubits being in nonequivalent quantum states in respect to the energy of qubits being in the equivalent quantum states. Such energy difference, 4 *K*, is determined by the coupling coefficient *g*. By making use of the instanton method of analysis^15^,^20^ we obtain that even in the case of a weak interaction, i.e. *K* ≪ Δ, a large amount of qubits 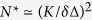 are synchronized, and these qubits display collective quantum-mechanical oscillations of a single frequency. Correspondingly, 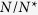 is a total number of diverse quantum oscillations observed in disordered SQMs. Here, *δ*Δ is the typical spread of energy level differences of individual qubits.

We show also that such quantum-mechanical synchronization phenomenon can be mapped to the Anderson localization^21^ of spinon-type excitations occurring in the SQM. Indeed, the various disordered SQMs with nearest-neighbor interactions can be described by the quantum Izing model with a large transverse magnetic field^22^,^23^. In the limit of *K* < Δ the ground state of this model is the spin ordering in the transverse direction, and the low-lying excited states form the spinon band separated from the ground state by the energy gap of order Δ. In the presence of disorder all spinon states are localized, and a spread of localized wave function of spinons is of order *r*_0_. Therefore, the coherent quantum mechanical oscillations correspond to the resonant transitions between the ground state and the various excited states of localized spinons.

We anticipate that the fabrication of disordered SQMs with a tunable inductive coupling allows one to observe the crossover from a non-synchronized regime to the synchronized regime, and provides the direct evidence of synchronized quantum-mechanical oscillations. The realization of synchronized regime in intrinsically disordered SQMs will result in a substantial simplification of the process of qubits addressing. Since arrays of interacting qubits are exactly mapped on various quantum spin models, our method of instanton analysis can be used for a quantitative theoretical study of low-lying quantum-mechanical states in diverse interacting disordered quantum systems under equilibrium and non-equilibrium conditions^22^,^23^,^24^,^25^.

## Results

### Superconducting Quantum Metamaterial Model and Hamiltonian of disordered SQM

**Figure 1:**
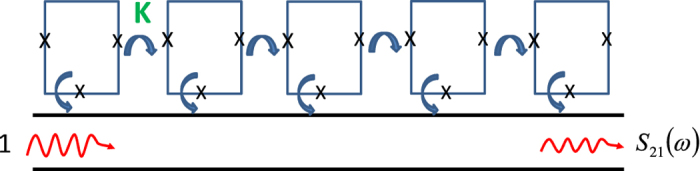
The schematic of an experimental setup allowing one to observe the coherent quantum-mechanical oscillations in the SQM. The SQM based on an array of flux qubits (3-Josephson junction SQUID) is shown. The array of qubits is coupled to the transmission line. The inductive coupling between adjacent qubits, *K*, is taken into account. The amplitude of transmitted electromagnetic wave is |*S*_21_|.

Let us to consider a one-dimensional array of *N* flux qubits modeled as two-level systems, with nearest-neighbor inductive electromagnetic interactions between adjacent qubits. In a particular case of flux qubits (3-Josephson junction SQUID) the two states correspond to the clockwise and anticlockwise flowing currents, and the energy level difference, Δ_*i*_, is determined by quantum tunneling between these states^12^. The frequencies of individual coherent quantum-mechanical oscillations that can be excited in a single qubit, are *ω*_*i*_ = Δ_*i*_/*ħ*. The SQM is coupled to the linear transmission line, and this setup allows one to study the propagation and reflection of electromagnetic waves through the SQM. The schematic of such setup is shown in Fig. 1. Notice here, that similar setup has been used in ref. 1 in order to measure macroscopic quantum-mechanical oscillations excited in the disordered SQM.

The quantum dynamics of each qubit is characterized by the imaginary-time dependent degree of freedom, *φ*_*i*_(*τ*), and the qubits Hamiltonian has a form:


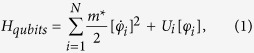


where both the parameter 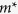 and the potentials *U*_*i*_[*φ*] determine completely quantum-mechanical dynamics and the energy levels of isolated qubits. Moreover, the double-well potential *U*_*i*_[*φ*] modelled as 
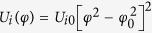
 results in small quantum-mechanical energy level differences Δ_*i*_ of single qubits. Here, ±*φ*_0_ are the values of *φ* in the classical stable states. In this model the energy level differences Δ_*i*_ are expressed as


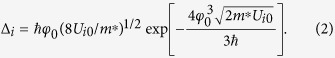


For flux qubits the parameters m^*^, *φ*_0_ and *U*_*i*0_ are determined by the capacitance and Josephson critical currents of Josephson junctions of qubits^12^. The unavoidable disorder in qubit parameters (*U*_*i*0_) leads to a spread of Δ_*i*_ in the SQM. The Hamiltonian of inductive electromagnetic interaction between adjacent qubits is written as


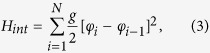


where the parameter *g* characterizing the strength of nearest-neighbor interaction is determined by mutual inductance between qubits. Thus, one can see that in the quantum regime the inductive interaction results in an increase of energy of qubits being in nonequivalent quantum states, 4 *K*, where 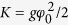 is the effective strength of interaction between adjacent qubits.

### Partition Function and Instanton Analysis

The thermodynamic properties of disordered SQMs are determined by the partition function *Z* expressed through the Feynman path integral in the imaginary-time *τ* representation as


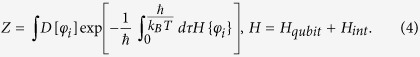


**Figure 2:**
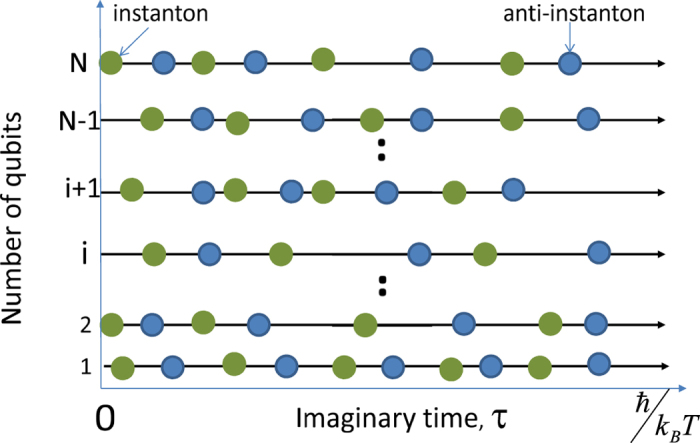
The schematic of a typical path configuration. It consists of alternating instanton (green circles) and anti-instanton (blue circles) solutions. The *τ* is the imaginary time. Each qubit is characterized by a total quantity of instantons and anti-instantons, *N*_*i*_.

We consider disordered SQMs with a weak electromagnetic interaction, i.e. *K* ≪ Δ_*i*_, and in this case the optimal path configurations are series of alternating instanton (anti-instanton) solutions uncorrelated in imaginary time interval [0, *ħ*/(*k*_*B*_*T*)]^15^,^20^ (schematic of a typical path configuration is shown in Fig. 2). For a single *i*-th qubit the optimal path configuration *φ(τ*) has a form:


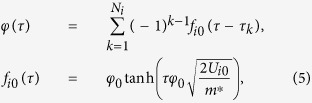


where *N*_*i*_ is the total number of instantons and (anti)instantons occurring on the time scale *ħ*/(*k*_*B*_*T*). In this approach the energy level differences Δ_*i*_ are obtained as


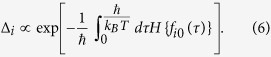


Since the probability density to obtain a single instanton in the interval *dτ* is determined by Δ_*i*_ the partition function *Z*_*i*_ of a single qubit is written as


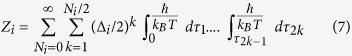


and 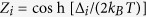. As the electromagnetic interaction between qubits is absent, i.e. *K* = 0, one can obtain 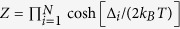, and on the time interval [0, *ħ*/(*k*_*B*_*T*)] the average quantity of instantons and anti-instantons for an *i*-qubit 〈*N*_*i*_〉 = Δ_*i*_/(*k*_*B*_*T*)^20^.

To analyze the collective behavior of disordered SQM in the presence of interaction we will characterize each qubit by random value of *N*_*i*_, and the probability *P*_*K*=0_{*N*_*i*_} shows sharp peaks on the values *N*_*i*_ = 〈*N*_*i*_〉 as


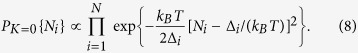


Moreover, in such description the frequencies *ω*_*i*_ of quantum-mechanical oscillations occurring in the SQM, are determined by random values of *N*_*i*_ as *ω*_*i*_ = *k*_*B*_*TN*_*i*_/*ħ*. Notice here, that in the presence of a weak (*K* ≪ Δ_*i*_) interaction between qubits the qubit frequencies *ω*_*i*_ can differ from bare frequencies, Δ_*i*_/*ħ*. Indeed, substituting instanton-(anti)instanton solutions (5) in the Eqs (3) and (4) we obtain the probability *P*_*K*_{*ω*_*i*_} in the following form:


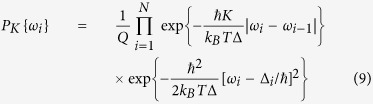


and the normalizing constant *Q* is expressed through the path integral as


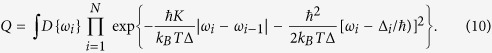


Here, 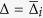 is the average value of energy splitting of qubits Δ_*i*_, and 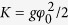 is the effective strength of electromagnetic interaction between instantons (anti-instantons) of adjacent qubits. We have taken into account that the spread of qubit splitting is small, i.e. *δ*Δ ≪ Δ. The physical meaning of different terms in Eq. (10) is rather transparent: the disorder in bare frequencies Δ_*i*_/*ħ* results in a spread of instanton quantities, *N*_*i*_ along the array, but the electromagnetic interaction allows to *equalize* the quantity of instantons on different qubits. Since the frequencies of quantum-mechanical oscillations are directly related to the quantities of instantons, one expect that the electromagnetic interaction results in the synchronization phenomena.

### Correlations of Qubits Frequencies

To analyze this effect quantitatively we consider the continuous limit as *N* ≫ 1, and using the periodic boundary conditions we expand *ω(x*) and Δ(*x*) (*x* is the coordinate along the array of qubits) in Fourier series as:


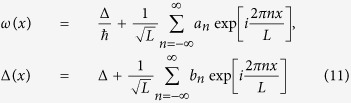


Here, *L* = *Na* is the length of the SQM, and *a* is the distance between qubits.

Taking into account that the values of Δ_*i*_ are not correlated for different qubits, and the characteristic spread of bare energy level differences is *δ*Δ we obtain |*b*_*n*_|^2^ = 2(*δ*Δ)^2^*a*. The coordinate dependent correlations in qubit frequencies are characterized by the correlation function 〈*ω(x*_1_)*ω(x*_2_)〉:


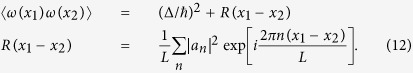


Thus, the distribution of qubit frequencies *ω(x*) is determined by the Fourier coefficients *a*_*n*_, which, in turn, can be obtained from the analysis of the partition function of interacting instanton liquid, *Q*. Substituting (11) in (10) the expression for Q is written in the following form:


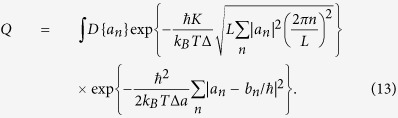


The optimal path *a*_*n*_ in the Eq. (13) is obtained by following procedure. By making use of the substitution 
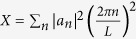
 and the equality 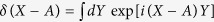, the Eq. (13) is transformed to the following form


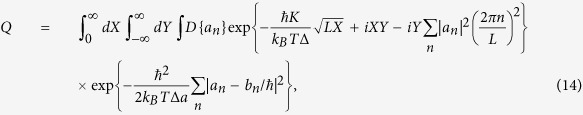


and calculating the Gaussian integrals over *a*_*n*_ explicitly we obtain


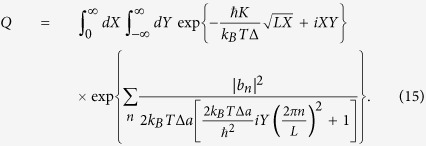


The maximum of the exponent in (15) is reached for optimal values of *X*_0_ and *Y*_0_, which are solutions of the system of equations


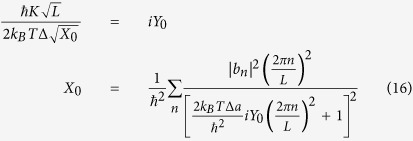


Taking into account that for the uncorrelated disorder in values of Δ_*i*_, the Fourier coefficients |*b*_*n*_|^2^ = 2(*δ*Δ)^2^*a*, one can obtain *iY*_0_ = 2*ħ*^2^*K*^4^*a*/[*k*_*B*_*T*Δ(*δ*Δ)^4^].

The dominant contribution to the the *Q* in (14) occurs from particular values of *a*_*n*_ which are determined by random values of qubit splitting *b*_*n*_. Explicitly the amplitudes *a*_*n*_ are obtained by maximizing the exponent in (14) over the variables *a*_*n*_ as


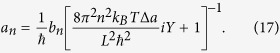


Substituting in this expression *Y* = *Y*_0_ the amplitudes *a*_*n*_ are written as


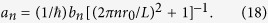


Here, we introduce the correlation radius *r*_0_ characterizing the area where the quantum-mechanical oscillations are correlated, i.e. 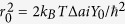.

In spite of the presence of uncorrelated disorder in qubits splitting, Δ_*i*_, the electromagnetic interaction between qubits can lead in long-range correlations (*r*_0_ ≫ *a*) of frequencies of quantum-mechanical oscillations excited in the disordered SQMs. These correlations are described quantitatively by coordinate-dependent correlation function of qubit frequencies *R(x*) (Equation (12)). Substituting the amplitudes *a*_*n*_ in (12) and calculating the sum over *n*, the correlation function *R(x*) is written explicitly as


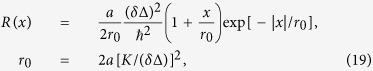


where the correlation radius *r*_0_ can greatly exceed the distance between qubits *a*. A peculiar dependence of correlation radius on the strength of disorder has an origin in a sub-diffusive interacting term ∝|*ω*_*i*_ − *ω*_*i*−1_| in the exponent of (10). It differs such a problem from e.g. fluctuation induced bending of strings and superconducting vortex lines^26^.

Next, we notice that this analysis can be extended to the two-dimensional lattice of interacting qubits. Indeed, the Eqs (16) are valid for 2*d* square lattice with the substitution: (1/*L*^2^)∑_*n*_ → ∫*qdq*. Calculating all integrals we obtain that in a 2d case the correlation radius is exponentially large, i.e. 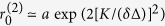.

The intrinsic correlations of frequencies, and, in particular, the dependencies of the correlation radius *r*_0_ on the strength of interaction *K* and the disorder *δ*Δ strongly resemble the phenomenon of Anderson localization in disordered low-dimensional solid state systems^21^. The origin of such similarity is the following: the disordered SQMs with nearest-neighbor interactions are mapped exactly on the quantum Izing model with a large transverse magnetic field^22^,^23^. The Hamiltonian of such a model is written as


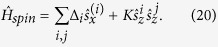


**Figure 3:**
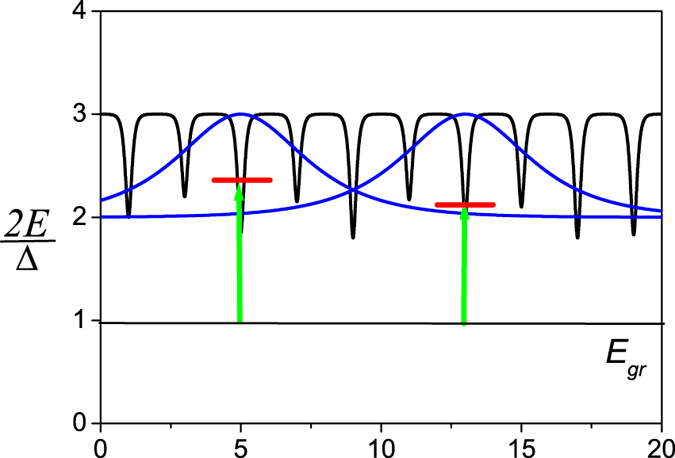
The schematic of the Anderson localization in the quantum Izing model with transverse magnetic field. Here, 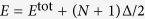, where *E*^tot^ is the total energy of the spin chain, and *N* is the total number of spins. The *x* is the coordinate along the spin chain, and *a* is the distance between spins. The ground state energy, 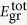 = −*N*Δ/2, the effective potential for the low-lying excited states (the spinon band of width *K*) are shown. The wave functions of excited states (blue lines) are localized, and a spread of spinon wave functions characterizes the number of quantum-mechanical oscillations in the SQM. These oscillations correspond to the resonant transitions (green lines) between the ground state and the excited states of localized spinons.

In the limit of *K* < Δ the ground state of this model is the spin ordering in the *x* direction, and the low-lying excited states form the *spinon band* of the width *K* separated from the ground state by the energy gap of order Δ. In the presence of disorder all spinon states are localized, and a spread of localized wave function of spinons is of order *r*_0_. Such Anderson localization in the spinon band is shown schematically in Fig. 3. Therefore, the coherent quantum mechanical oscillations correspond to the resonant transitions between the ground state and the various excited states of localized spinons.

### Electromagnetic Waves Transmission through the SQM

The coherent quantum-mechanical oscillations excited in the SQM can be observed through the frequency dependent transmission coefficient |*S*_21_(*ω*)|^2^ of electromagnetic waves propagating in the transmission line inductively coupled to the SQM^1^,^17^. The |*S*_21_(*ω*)|^2^ shows a set of resonant dips whose positions are determined by resonant conditions *ω* = *ω*_*i*_. Quantitatively, it can be described as refs 15 and 16


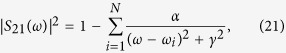


where the parameter *α* ≪ *γ*^2^ characterizes the coupling between the transmission line and SQM. Thus, as the electromagnetic interaction between adjacent qubits are small, i.e. *K* ≪ *δ*Δ, and a substantial spread of qubit splitting *δ*Δ ≫ *γ*, the disordered SQM supports *N* non-synchronized coherent quantum oscillations of different frequencies. These non-synchronized coherent quantum-mechanical oscillations manifest themselves by *N* small resonant dips of magnitude 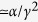 in the |*S*_21_(*ω*)|^2^ (see, Eq. (21)).

However, the crossover to partially synchronized regime occurs as the inductive interaction between nearest-neighbors qubits overcomes the disorder in qubits energy level differences, i.e. *K* ≥ *δ*Δ. In this regime the correlation radius *r*_0_ exceeds the distance *a* between qubits, and a large amount of qubits, 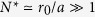 displays collective synchronized behavior characterized by a single frequency. In this limit the |*S*_21_|^2^ displays 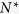 dips of large magnitude 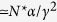. As the interaction *K* goes over the value (*δ*Δ)*N*^1/2^ all qubits become synchronized, the collective quantum mechanical oscillations are established in a whole SQM, and a single large dip occurs in the |*S*_21_|^2^. Notice here, that main assumption of our analysis is the absence of correlations of instanton (anti-instanton) positions on the *τ*-axis, and this assumption is valid as *K* < Δ.

## Discussion

In conclusion we have theoretically analyzed the excitation of coherent quantum-mechanical oscillations in disordered SQMs. Our analysis is based on the mapping of coherent quantum-mechanical oscillations to series of alternating instanton (anti-instanton) solutions in the path-integral approach. In this model the frequencies of quantum-mechanical oscillations excited in the SQMs, *ω*_*i*_, are directly related to the quantity of instantons on different qubits, *N*_*i*_. The disorder in qubits parameters results in a spread of *N*_*i*_ along the array of qubits, and a weak electromagnetic interaction between adjacent qubits, *K*, leads to the partial alignment of these quantities, *N*_*i*_. Thus, we have obtained that a large amount of qubits can display synchronized collective behavior characterized by a single frequency. The size of the region showing synchronized behavior is determined by the ratio of the strength of interaction *K* to the typical spread of energy splitting of qubits, *δ*Δ.

The fingerprints of synchronized regimes of coherent quantum oscillations are a few number of giant resonant dips in the frequency dependent electromagnetic waves transmission, |*S*_21_(*ω*)|^2^ (see Fig. 1, and Eq. (21)). In the synchronized quantum-mechanical dynamic state a number of such resonant dips is 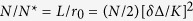. In ref. 1 the propagation of electromagnetic waves through the SQM containing of 20 qubits has been experimentally studied. In these experiments one or two giant resonant dips in the frequency dependent transmission coefficient, |*S*_21_(*ω*)|^2^, have been observed. These results indicate the excitation of synchronized coherent quantum mechanical oscillations in the SQM, and a plausible qualitative explanation of these results is the presence of substantial effective inductive coupling between adjacent qubits, i.e. 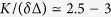. However, one can not exclude an alternative explanation that a long-range interaction in an array of qubits also can induce the synchronized quantum-mechanical dynamic state. Such an interaction originates from the emission (absorption) of virtual photons of a low-dissipative resonator^2^,^15^,^19^. This type of interaction leads to the interaction between instantons (anti-instantons) of well separated qubits, and it also allows to equalize quantities of instantons (anti-instantons) on different qubits, and therefore, to establish a synchronized regime. We will address the quantitative analysis of synchronized quantum-mechanical oscillations induced by long-range interaction, elsewhere.

## Additional Information

**How to cite this article**: Fistul, M. V. Quantum synchronization in disordered superconducting metamaterials. *Sci. Rep.* **7**, 43657; doi: 10.1038/srep43657 (2017).

**Publisher's note:** Springer Nature remains neutral with regard to jurisdictional claims in published maps and institutional affiliations.
